# Human umbilical cord-derived mesenchymal stem cells/Bio-Oss granules composite prevents medication-related osteonecrosis of the jaw in a rat model

**DOI:** 10.3389/fbioe.2026.1800764

**Published:** 2026-04-24

**Authors:** Zhi Chen, Keying Shi, Letian Shan, Jing Zhao, Le Guo, Liwen Wang, Zheng He, Yuanna Zheng

**Affiliations:** 1 School/Hospital of Stomatology, Zhejiang Chinese Medical University, Hangzhou, Zhejiang, China; 2 Ningbo Dental Hospital/Ningbo Oral Health Research Institute, Ningbo, Zhejiang, China; 3 The First Affiliated Hospital of Zhejiang Chinese Medical University, Hangzhou, Zhejiang, China; 4 Yichen Biotechnology Co., Ltd, Hangzhou, Zhejiang, China

**Keywords:** Bio-Oss granules, bone regeneration, human umbilical cord-derived mesenchymal stem cells, medication-related osteonecrosis of the jaw, osteogenic differentiation

## Abstract

**Objective:**

To evaluate the preventive efficacy of human umbilical cord-derived mesenchymal stem cells (hucMSCs)/Bio-Oss granules composite in a rat model of medication-related osteonecrosis of the jaw (MRONJ).

**Materials and methods:**

Twenty-four rats were randomly allocated into a negative control group, a model control group, a Bio-Oss granules group, and a composite group (n = 6). All groups except the negative control group received intraperitoneal injections of zoledronic acid (0.1 mg/kg) 3 times per week for 12 weeks to induce MRONJ-like lesions. After 4 weeks of drug administration, standardized bone defects were prepared at the mandibular molar extraction sites. In the negative control and model control groups, the defects were left unfilled. In the Bio-Oss granules and composite groups, the defects were filled with Bio-Oss granules or hucMSCs/Bio-Oss granules composite, respectively. All rats were euthanized 8 weeks after surgery. Macroscopic observation, micro-CT evaluation, and histological analysis were conducted to assess bone exposure rate, bone volume fraction (BV/TV), bone mineral density (BMD), and the percentage of empty osteocyte lacunae.

**Results:**

The bone exposure rate was 0% in both the negative control and composite groups, compared with 83.33% and 66.67% in the model control and Bio-Oss granules groups, respectively. The composite group showed improved mucosal coverage, as well as significantly higher BV/TV and BMD and fewer empty osteocyte lacunae than the other bisphosphonate (BP)-treated groups (*P* < 0.001).

**Conclusion:**

Bio-Oss granules alone showed limited efficacy in preventing MRONJ. In contrast, the hucMSCs/Bio-Oss granules composite effectively promoted mucosal healing and bone regeneration, thereby preventing the development of MRONJ in BP-treated rats.

## 1 Introduction

Medication-related osteonecrosis of the jaw (MRONJ) is a severe complication associated with antiresorptive and antiangiogenic therapies (Marx, 2003; Ruggiero et al., 2022). Tooth extraction is the major predisposing factor, often resulting in exposed necrotic bone and impaired wound healing (Ruan et al., 2024a). Current therapeutic outcomes for MRONJ remain unsatisfactory. Conservative treatments often show limited efficacy, while surgical interventions, although more effective, are invasive and associated with functional impairment and recurrence (Rupel et al., 2014; Ruan et al., 2024b). Although various adjunctive approaches have been explored, their clinical application remains limited due to insufficient evidence (Kaibuchi et al., 2021). Therefore, there is a pressing and unmet need for novel, effective strategies to prevent and treat MRONJ.

The pathogenesis of MRONJ is not yet fully elucidated, and impaired bone remodeling is considered one of the central mechanisms (Jiang et al., 2023). Bisphosphonates (BPs) have been shown to disrupt the function of osteoclasts, osteoblasts, and mesenchymal stem cells (MSCs), thereby disturbing bone homeostasis (Abe et al., 2012; Ogata et al., 2015; He et al., 2017). Increasing evidence suggests that MSC dysfunction plays a critical role in the development of MRONJ. MSCs derived from MRONJ lesions exhibit impaired proliferation, self-renewal, and differentiation, along with a reduced capacity to support osteoclastogenesis, leading to defective bone remodeling and delayed healing (He et al., 2017). These findings highlight the potential of MSC-based approaches for the prevention and treatment of MRONJ. In parallel, recent studies have explored advanced biomaterial-based and nano-enabled strategies to enhance tissue regeneration and modulate the local microenvironment (Wang et al., 2024; Kong et al., 2025), further supporting the rationale for combining cells with functional biomaterials. Previous studies have demonstrated that MSC-based composite systems using biomaterial scaffolds can promote healing in MRONJ models. Rodríguez-Lozano et al. (2020) demonstrated that β-TCP combined with bone marrow-derived MSCs applied to extraction sockets in BP-treated rats effectively prevented MRONJ onset. Likewise, Zang et al. (2019) reported favorable outcomes using coral-derived hydroxyapatite (HA) scaffolds loaded with adipose-derived MSCs in an MRONJ-like rabbit model. However, these approaches primarily rely on MSC sources that require invasive harvesting procedures and on biomaterial scaffolds that are not widely used in clinical practice, which may limit their direct clinical translation.

Among the various MSC sources, human umbilical cord-derived MSCs (hucMSCs) have attracted increasing attention due to their high proliferative capacity, robust differentiation potential, and low immunogenicity (Cho et al., 2007; Hsieh et al., 2010). This low immunogenicity is associated with a reduced risk of immune rejection following transplantation (Cho et al., 2007; Feng et al., 2022). In addition, hucMSCs can be obtained non-invasively from discarded perinatal tissues, avoiding the limitations associated with invasive harvesting procedures required for bone marrow- or adipose-derived MSCs (Liao and Chen, 2014). These features make hucMSCs a promising and clinically relevant cell source for regenerative applications.

Bio-Oss, a widely used deproteinized bovine bone substitute, is well known for its excellent osteoconductive properties and clinical applicability (Lee et al., 2019; Iaquinta et al., 2022). Our previous study also demonstrated that a platelet-rich plasma/Bio-Oss granules composite effectively prevented MRONJ in BP-treated rats (Shi et al., 2025). Therefore, Bio-Oss may serve as a clinically relevant scaffold for MSC-based strategies. However, studies investigating the combination of MSCs with Bio-Oss in MRONJ remain limited, and its therapeutic potential warrants further investigation.

In this study, we aimed to evaluate the preventive efficacy of a hucMSCs/Bio-Oss granules composite against MRONJ in BP-treated rats. The composite was applied to bone defect sites to assess its potential as a novel preventive strategy. We hypothesized that the model control group would exhibit impaired healing compared with the negative control group, and that treatment with Bio-Oss granules or the hucMSC/Bio-Oss granules composite might improve healing outcomes, with the composite group showing superior regenerative effects, approaching normal levels.

## 2 Materials and methods

### 2.1 Culture and characterization of hucMSCs

**FIGURE 1:**
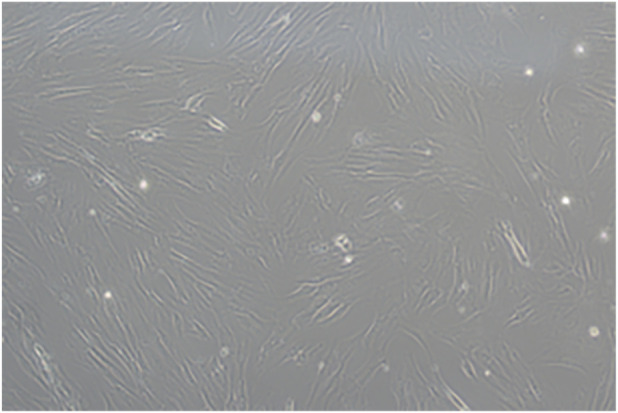
Microscopic morphology of human umbilical cord-derived mesenchymal stem cells (hucMSCs) observed under ×10 magnification.

Primary hucMSCs (Hangzhou Sanjiang Shangyu Biotechnology, China) were cultured in alpha-minimum essential medium (α-MEM; Gibco, United States) supplemented with 10% fetal bovine serum (Gibco, United States) at 37 °C in a humidified atmosphere containing 5% CO_2_. The culture medium was replaced every 3 days. When the cells reached approximately 90% confluence, they were passaged using 0.25% trypsin (Biosharp, China). Third-passage hucMSCs were used for all subsequent experiments (Figure 1).

For immunophenotypic characterization, hucMSCs were analyzed by flow cytometry in accordance with the criteria established by the International Society for Cellular Therapy (ISCT, Dominici et al., 2006). Briefly, cells were harvested at a density of 1 × 10^6^ cells per tube, incubated with antibodies against CD34, CD73, CD90, and CD105 at 4 °C for 30 min in the dark, with an isotype control included to define background fluorescence and establish gating thresholds, and subsequently analyzed using a flow cytometer (Thermo Fisher, United States) to determine the expression of surface markers.

### 2.2 DAPI staining and DNA/RNA quantification of Bio-Oss granules

To verify that Bio-Oss granules contained negligible nucleic acids that would not interfere with subsequent qRT-PCR analysis, DAPI staining and DNA/RNA quantification were performed. Bio-Oss granules (Geistlich, Switzerland) was fixed on glass slides with 4% paraformaldehyde (Biosharp, China) for 15 min, washed 3 times with PBS, and incubated with DAPI (10 μg/mL; Sigma, United States) for 15 min at room temperature under light-protected conditions. Following 3 washes of 5 min each with PBS, the samples were mounted and examined under a fluorescence microscope (Nikon, Japan).

For nucleic acid quantification, DNA and RNA were extracted separately from 20 mg Bio-Oss granules. DNA was isolated using the DNeasy Blood & Tissue Kit (Qiagen, Germany), whereas RNA was extracted using TRIzol reagent (Thermo Fisher, United States), both according to the manufacturers’ protocols. The concentrations of DNA and RNA were determined using a NanoDrop 2000 spectrophotometer (Thermo Fisher, United States). All measurements were performed in triplicate, and mean values were calculated.

### 2.3 qRT-PCR analysis of hucMSCs/Bio-Oss granules co-culture system

To characterize the expression of osteogenic and other lineage- and stemness-associated genes in hucMSCs under co-culture conditions with Bio-Oss granules, real-time quantitative polymerase chain reaction (qRT-PCR) analysis was performed. HucMSCs were seeded under sterile conditions into low-attachment 6-well plates (Thermo Fisher, United States) at a density of 5 × 10^4^ cells per well and cultured in α-MEM supplemented with 10% fetal bovine serum at 37 °C in a humidified atmosphere containing 5% CO_2_. For the co-culture condition, Bio-Oss granules (0.1 g/well) were placed in the wells prior to cell seeding to allow direct contact between hucMSCs and the biomaterial. Cells cultured without Bio-Oss granules served as the control group. The use of low-attachment plates minimized preferential cell adhesion to the plastic surface and facilitated cell-material interaction during co-culture.

At days 7, 14, and 21, total RNA was extracted using the TRIzol reagent according to the manufacturer’s instructions. Complementary DNA was synthesized from the extracted RNA using the HiScript II Q RT SuperMix for qPCR kit (Vazyme, China). Subsequently, qRT-PCR was performed with primers specific for Runt-related transcription factor 2 (RUNX2), alkaline phosphatase (ALP), osteopontin (OPN), aggrecan (ACAN), SRY-box transcription factor 9 (SOX9), collagen type II alpha 1 chain (COL2A1), collagen type X alpha 1 chain (COL10A1), peroxisome proliferator-activated receptor gamma (PPARγ), and octamer-binding transcription factor 4 (OCT4). Glyceraldehyde-3-phosphate dehydrogenase (GAPDH) was used as the housekeeping gene and internal control. Relative gene expression levels were calculated using the ^ΔΔ^Ct method. All experiments were performed in triplicate. Primer sequences of the target genes are listed in Table 1.

**TABLE 1:** Primer sequences used for real-time PCR analysis.

Gene	Forward primer	Reverse primer
*RUNX2*	CAC​TGG​CGC​TGC​AAC​AAG​A	CAT​TCC​GGA​GCT​CAG​CAG​AAT​AA
*ALP*	ATG​GAG​AGC​AAA​GCC​CTG​CTC	GTT​AGG​TCC​AGC​TGG​ATC​GAG
*OPN*	TTG​CAG​TGA​TTT​GCT​TTT​GC	CAT​CGT​CAT​GGC​TTT​CAT​TG
*ACAN*	GCC​TCA​GGA​CTT​CCA​GAA​A	CAC​TGG​ACT​CAA​CAA​GCT​G
*SOX9*	GGG​CTG​CCT​AAA​TTG​TGT​GT	AGC​AAC​CCT​CAA​ACT​CTC​CA
*COL2A1*	GAA​CCC​AGA​AAC​AAC​ACA​ATC​C	GTT​CGG​ACT​TTT​CTC​CCC​TC
*COL10A1*	CTG​AGC​GAT​ACC​AAA​CAC​C	GTA​AAG​GTG​TAT​CAC​TGA​GAG​G
*PPARγ*	TCAGGTTTGGGCGAATG	TTTGGTCAGCGGGAAGG
*OCT4*	GAC​AAC​AAT​GAG​AAC​CTT​CAG​GAG​A	CTG​GCG​CCG​GTT​ACA​GAA​CCA
*GAPDH*	CGG​GCG​TTG​ATG​ACA​AGT​TTC​CCG	CTA​CCC​ACG​GCA​AAT​TCC​AC

### 2.4 Preparation of hucMSCs/Bio-Oss granules composite

**FIGURE 2:**
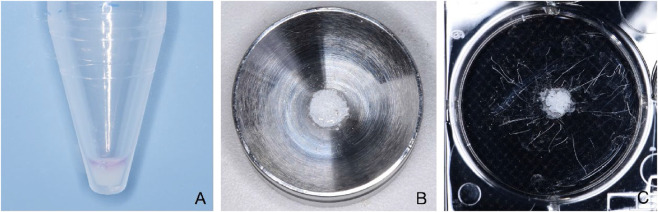
Preparations of hucMSCs/Bio-Oss granules composite. **(A)** Cell pellet of third-passage hucMSCs after centrifugation. **(B)** Bio-Oss granules. **(C)** hucMSCs/Bio-Oss granules composite.

Third-passage hucMSCs were centrifuged using a 5804R refrigerated centrifuge (Eppendorf, Germany) at 1000 rpm for 10 min, and the supernatant was discarded (Figure 2A). The cell pellet was resuspended in DMEM medium (Cienry, China), and the concentration was adjusted to 1 × 10^10^ cells/L. Bio-Oss granules (Figure 2B) were weighed on an electronic balance and aliquoted into 6-well plates at 0.01 g per well. A 3 μL suspension of hucMSCs was added to each portion of bone granules to ensure complete immersion. The constructs were incubated at 37 °C in a 5% CO_2_ incubator for 4 h. Subsequently, the composites (Figure 2C) were transferred to the operating room for implantation.

### 2.5 Transplantation of materials in bone defect sites

Twenty-four female Sprague-Dawley rats (200–250 g, 6–8 weeks old) were used in this study. All animal procedures were approved by the Institutional Animal Care and Use Committee of Zhejiang Chinese Medical University (Approval No. IACUC-20220613-09) and were conducted in accordance with the National Institutes of Health guidelines for the care and use of laboratory animals. The animals were randomly allocated into four groups (n = 6 per group): (1) negative control group (no BP treatment and no material filling), (2) model control group (zoledronic acid treatment without material filling), (3) Bio-Oss granules group (zoledronic acid treatment with Bio-Oss granules filling), and (4) composite group (zoledronic acid treatment with composite material filling). Outcome assessment and data analysis were performed in a blinded manner whenever possible to minimize potential bias. To adhere to the principles of animal welfare and reduce unnecessary animal use, the model control group and the Bio-Oss granules group were shared with a parallel study conducted within the same research project (Shi et al., 2025). Specifically, the same animals were used in both studies, and the corresponding data have been previously reported and are shared across studies. The negative control group represented physiological healing conditions, receiving saline injections instead of zoledronic acid and leaving the bone defects unfilled.

Given the exploratory nature of the current study, sample size was determined using the resource equation method (Charan and Kantharia, 2013), with consideration of ethical and practical aspects of animal use. According to this approach, the value E (defined as the total number of animals minus the total number of groups), which represents the degrees of freedom for analysis of variance (ANOVA), should lie between 10 and 20 to ensure an adequate sample size. In the present study, E = 24–4 = 20, which falls within the recommended range, indicating that the selected sample size was appropriate.

**FIGURE 3:**
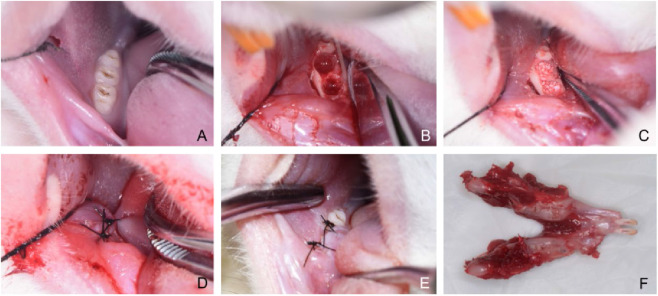
Surgical procedures of the mandible in Sprague–Dawley rats. **(A)** Pre-extraction view of the mandibular right first and second molars. **(B)** Creation of standardized bone defects at the extraction sockets. **(C)** Filling of the defects with Bio-Oss granules or hucMSC/Bio-Oss composite material. **(D)** Closure of the gingiva with 6–0 non-absorbable sutures. **(E)** Suture removal 1 week after surgery. **(F)** Harvesting and dissection of the mandible at 8 weeks postoperatively.

All animals were subjected to a 1-week adaptive feeding. Rats in the model control, Bio-Oss granules, and composite groups received intraperitoneal injections of zoledronic acid (Novartis, Switzerland) at a dose of 0.1 mg/kg (0.8 mg/mL, 0.125 mL/kg), whereas rats in the negative control group were administered an equivalent volume of 0.9% saline (0.125 mL/kg; Otsuka, China). Injections were performed 3 times per week. After 4 weeks of drug administration, all groups underwent surgery, followed by continued injections at the same dose and frequency for an additional 8 weeks. Anesthesia was induced by intraperitoneal injection of Zoletil (22.5 mg/mL, 2 mL/kg; Virbac, France). Surgical procedures were performed after adequate anesthesia was achieved. After disinfection of the surgical field, a temporary cheek traction suture was applied to improve surgical visualization and was removed immediately after the procedure. The gingiva was separated using a small curette, the mandibular right first and second M were loosened with a sharp dental probe, and then extracted with hemostatic forceps, leaving the third molar intact (Figure 3A). Subsequently, bone defects were prepared at the extraction sockets of the first and second M using a slow-speed dental handpiece equipped with 1.4 mm and 1.8 mm round burs, respectively, until the bur passed beyond the alveolar crest. During drilling, sterile saline irrigation was applied for cooling. This procedure created 2 bone defects with a volume of approximately 3.8 mm^3^ each (Figure 3B).

In the negative control and model control groups, the bone defects were left unfilled after thorough cleaning. In the Bio-Oss granules group, the defects were filled with Bio-Oss bone granules (0.01 g) until flush with the alveolar crest. In the hucMSC/Bio-Oss composite group, the defects were filled with hucMSC/Bio-Oss granules composite (3 × 10^4^ hucMSCs per 0.01 g Bio-Oss granules) until flush with the alveolar crest (Figure 3C). Finally, the gingiva was closed with 6–0 non-absorbable sutures (Ethicon, United States) in all groups (Figure 3D). The sutures were removed 1 week after surgery (Figure 3E). No antibiotics or analgesics were administered postoperatively in order to avoid potential confounding effects on inflammatory responses and bone healing processes and to facilitate the establishment of the MRONJ model (Ruggiero et al., 2022; Chuang et al., 2024).

At 8 weeks postoperatively, all rats were euthanized by carbon dioxide inhalation. The mandibles were harvested, rinsed with saline, and fixed in 4% paraformaldehyde (Figure 3F).

### 2.6 Macroscopic observation

At 8 weeks postoperatively, the surgical sites in the right mandible of each rat were examined macroscopically to evaluate the presence of unhealed mucosa, exposed necrotic bone, or other abnormalities.

### 2.7 Micro-CT analysis

Mandibular specimens were scanned using a SkyScan Micro-CT system (Bruker, Germany) with an isotropic voxel size of 16 μm. The acquired data were reconstructed into three-dimensional (3D) images using NRecon software (Bruker, Germany) and subsequently analyzed with Imalytics Preclinical software (Gremse-IT, Germany). The regions corresponding to the surgically created bone defects in the right mandible were defined as regions of interest (ROI). ROIs were standardized to encompass the extraction sockets of the first and second M, corresponding to the defect sites created during surgery, and were applied consistently across all samples. The bone volume fraction (BV/TV) and bone mineral density (BMD) were calculated as the average values of the 2 ROIs to evaluate new bone formation.

### 2.8 Histological analysis

After micro-CT scanning, the mandibles were processed for histological analysis. The specimens fixed in paraformaldehyde were washed in running water to remove residual fixative, dehydrated in graded concentrations of ethanol, cleared in xylene, and then embedded in methyl methacrylate containing 15% dibutyl phthalate and 1% bis(tert-butyldioxyisopropyl)benzene. The specimens were cut buccolingually at the bone defect sites and sectioned to a thickness of 100 μm using an SP1600 saw microtome (Leica, Germany), followed by hematoxylin–eosin (H&E) staining (Solarbio, China). Histological evaluation was conducted under an optical microscope (Nikon, Japan). Mucosal continuity at the surgical site was examined at ×40 magnification. The percentage of empty osteocyte lacunae was quantified at ×100 magnification. For each specimen, 4 representative fields of view were randomly selected, and 50 osteocyte lacunae (including both empty and non-empty) were counted per field. Osteocyte lacunae without visible nuclei were defined as empty. The percentage of empty osteocyte lacunae was calculated as follows:

$$\text{Empty osteocyte lacunae }\left(\%\right)=\frac{\text{Number}\;\text{of}\;\text{empty}\;\text{osteocyte}\;\text{lacunae}}{\text{Total}\;\text{number}\;\text{of}\;\text{osteocyte}\;\text{lacunae}}\times 100$$

### 2.9 Outcome measures and definition of prevention

The primary outcome for evaluating the preventive efficacy of the Bio-Oss granules and hucMSCs/Bio-Oss granules composite was the absence of exposed bone at the surgical site at 8 weeks postoperatively, as assessed by macroscopic examination. Secondary outcome measures included histological evaluation of mucosal continuity and the percentage of empty osteocyte lacunae, as well as BV/TV and BMD assessed by micro-CT. Preventive success was defined as the absence of exposed bone at the surgical site, together with histological and radiological evidence of improved mucosal healing and bone regeneration, and a reduced percentage of empty osteocyte lacunae compared with the model control group.

### 2.10 Statistical analysis

Statistical analyses were performed using SPSS 26.0 software (IBM, United States). Quantitative data were expressed as the mean ± standard deviation. The Shapiro–Wilk test was used to examine the normality of data distribution. Comparisons between two groups (mRNA expression levels quantified by qRT-PCR) were performed using the independent-samples t-test. Categorical data (bone exposure rate) were analyzed using Fisher’s exact test for overall group comparisons, and pairwise comparisons were adjusted using the Bonferroni correction method (adjusted α = 0.008). Comparisons among multiple groups (BMD, BV/TV, and the percentage of empty osteocyte lacunae) were performed using one-way ANOVA followed by Bonferroni correction. A *P*-value <0.05 was considered statistically significant.

## 3 Results

**FIGURE 4:**
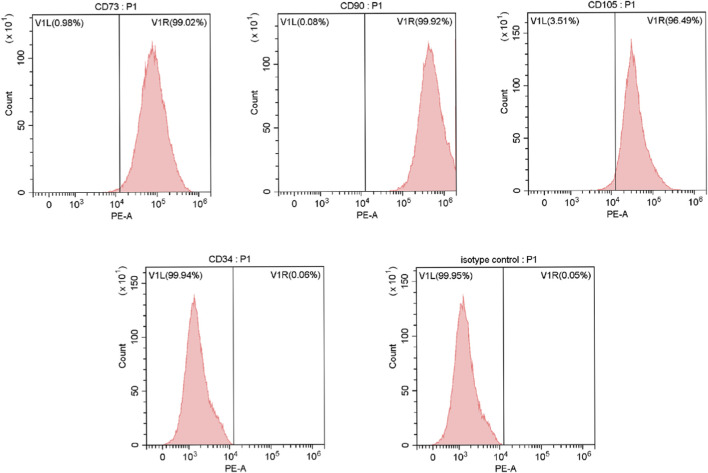
Flow cytometric characterization of hucMSCs surface markers (CD73, CD90, CD105, and CD34) with isotype control.

**FIGURE 5:**
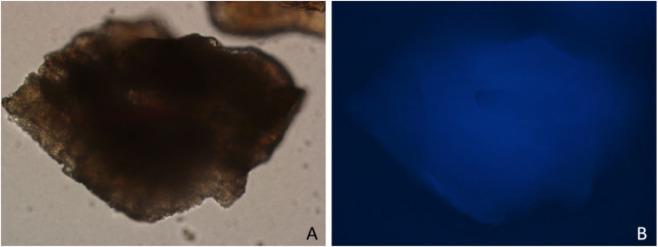
DAPI staining of Bio-Oss granules observed under fluorescence microscopy at ×100 magnification. **(A)** Bright-field image. **(B)** DAPI fluorescence image.

In the present study, the cultured hucMSCs highly expressed the surface markers CD73 (99.02%), CD90 (99.92%), and CD105 (96.49%), while showing low expression of CD34 (0.06%), consistent with the identification criteria for MSCs established by ISCT (Figure 4). Fluorescence microscopy after DAPI staining revealed no detectable DAPI-positive nuclei in the Bio-Oss granules, indicating minimal residual cellular components (Figure 5). Consistently, nucleic acid quantification showed that the concentrations of DNA and RNA extracted from the Bio-Oss granules were 5.30 ± 0.35 ng/μL and 4.17 ± 0.32 ng/μL, respectively, indicating low levels of nucleic acid content within the material.

**FIGURE 6:**
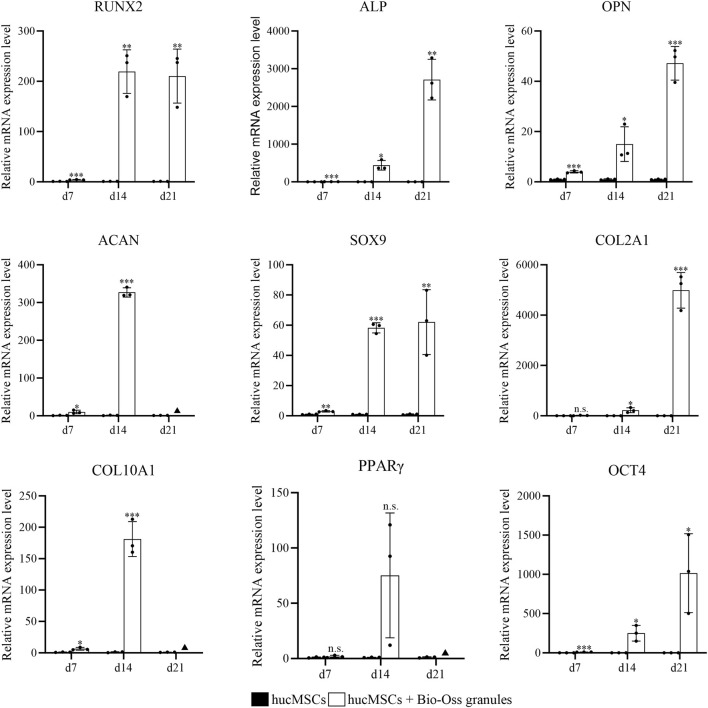
qRT-PCR analysis of mRNA expression levels of osteogenic-, chondrogenic-, adipogenic-, and stemness-related genes in hucMSCs cultured alone or co-cultured with Bio-Oss granules. The analyzed genes included osteogenic markers (RUNX2, ALP, OPN), chondrogenic markers (ACAN, SOX9, COL2A1), hypertrophic cartilage marker (COL10A1), adipogenic marker (PPARγ), and stemness marker (OCT4). Relative expression levels were calculated using the ΔΔCt method and are presented as mean ± SD (n = 3). *, *P* < 0.05; **, *P* < 0.01; ***, *P* < 0.001; n. s., not significant; ▲, gene expression not detected (independent-samples t-test).

qRT-PCR analysis revealed that hucMSCs co-cultured with Bio-Oss granules exhibited distinct gene expression profiles compared with hucMSCs cultured alone (Figure 6). The mRNA expression levels of osteogenic-related genes RUNX2, ALP, and OPN were significantly higher in the co-culture condition than in hucMSCs cultured alone at all examined time points (days 7, 14, and 21; *P* < 0.05). Regarding chondrogenic-related genes, ACAN expression was significantly increased at days 7 and 14 (*P* < 0.05), and SOX9 expression was markedly higher under co-culture at all time points (*P* < 0.01). COL2A1 expression showed no significant difference at day 7 (*P* > 0.05) but was significantly upregulated at days 14 and 21 (*P* < 0.05). The expression of COL10A1, a hypertrophic cartilage-related marker, was also significantly upregulated at days 7 and 14 (*P* < 0.05). For the adipogenic-related gene PPARγ, no significant difference was observed between the two culture conditions at days 7 and 14 (*P* > 0.05). In contrast, the stemness-related gene OCT4 exhibited significantly higher expression in the co-culture system at all examined time points (*P* < 0.05). Notably, at day 21, the mRNA expression of ACAN, COL10A1, and PPARγ in the co-culture condition was not detected under the current qRT-PCR conditions and was therefore not subjected to statistical analysis.

**FIGURE 7:**
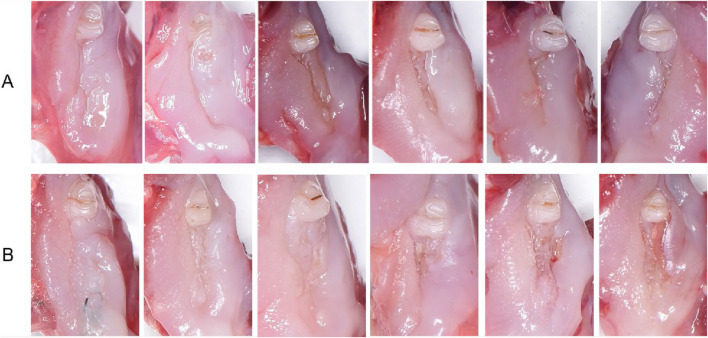
Macroscopic observations at 8 weeks post-surgery (n = 6). **(A)** Negative control group with satisfactory mucosal healing and no bone exposure. **(B)** Composite group with no bone exposure (0%, 0/6) and incomplete mucosal healing (33.3%, 2/6).

Macroscopic observations of the surgical sites at 8 weeks post-surgery are shown in Figure 7. In a few samples, minor residual suture fragments or debris (e.g., litter particles or hairs) were observed; however, these findings were limited and did not interfere with the overall evaluation of mucosal healing and bone exposure. In the negative control group, mucosal healing was satisfactory, and no exposed bone was observed (Figure 7A). In contrast, most rats in the model control and Bio-Oss granules groups displayed bone exposure without mucosal soft tissue coverage, resulting in bone exposure rates of 83.33% (5/6) and 66.67% (4/6), respectively, as previously reported (Shi et al., 2025). Conversely, the composite group showed a bone exposure rate of 0% (0/6), with complete mucosal healing observed in 66.67% (4/6) of the rats (Figure 7B). A significant difference in bone exposure rates was found among the groups (*P* < 0.05). However, pairwise comparisons with Bonferroni correction showed no significant differences between any two groups (all *P* > 0.008, Table 2).

**TABLE 2:** Comparison of bone exposure rates among groups.

Group	N	Bone exposure (n/N, %)	P value (overall)
Negative control	6	0/6 (0.00%)	0.003
Model control	6	5/6 (83.33%)	
Bio-Oss granules	6	4/6 (66.67%)	
Composite	6	0/6 (0.00%)	

Overall comparisons were performed using Fisher’s exact test. Pairwise comparisons were adjusted using the Bonferroni correction (adjusted α = 0.008) and were not statistically significant. Data for the model control and Bio-Oss granules groups were previously reported (Shi et al., 2025) and are included here for comparison

**FIGURE 8:**
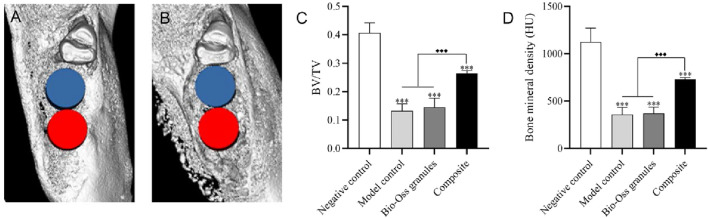
Micro-CT analysis at 8 weeks post-surgery (n = 6). **(A)** Representative three-dimensional reconstruction image of surgical site in negative control group. **(B)** Representative three-dimensional reconstruction image of surgical site in composite group. **(C)** Quantitative analysis of BV/TV in regions of interest (ROIs) **(D)** Quantitative analysis of bone mineral density (BMD) in ROIs. Data for the model control and Bio-Oss granules groups were previously reported (Shi et al., 2025) and are included here for comparison. ***, *P* < 0.001 vs. negative control group; ◆◆◆, *P* < 0.001 among the model control, Bio-Oss granules, and composite groups (one-way ANOVA with Bonferroni *post hoc* test).

Micro-CT analysis revealed the bone regeneration status within the bone defect sites at 8 weeks post-surgery (Figure 8). Representative 3D reconstruction images revealed extensive new bone regeneration within the defect sites in the negative control group (Figure 8A). In contrast, both the model control and Bio-Oss granules groups showed evident bone resorption around the ROIs, as previously reported (Shi et al., 2025). Notably, in the composite group, substantial new bone formation was also observed surrounding the bone defect sites (Figure 8B). The negative control group showed the highest BV/TV value (0.41 ± 0.04), which was significantly greater than those of the other three groups (*P* < 0.001). The BV/TV values of the model control group (0.13 ± 0.03) and Bio-Oss granules group (0.14 ± 0.03), were comparable (*P* > 0.05), whereas the composite group exhibited a significantly higher BV/TV (0.26 ± 0.10) than both (*P* < 0.001, Figure 8C). BMD analysis showed a similar trend. The negative control group had the highest BMD (1123.46 ± 148.78 HU), which was significantly greater than those of the other three groups (*P* < 0.001). The composite group exhibited a significantly higher BMD (731.92 ± 17.32 HU) than the model control group (359.07 ± 73.58 HU) and Bio-Oss granules group (370.79 ± 66.30 HU, *P* < 0.001), while no significant difference was observed between the latter two (*P* > 0.05, Figure 8D).

**FIGURE 9:**
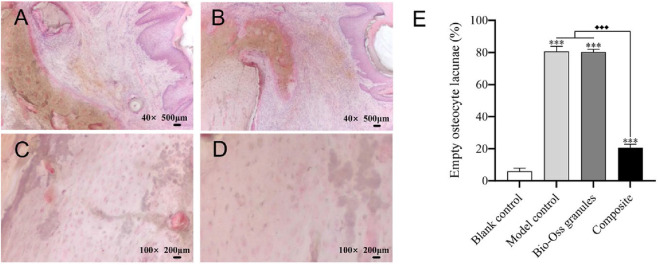
Histological analysis (HE staining) at 8 weeks post-surgery (n = 6). **(A,B)** Representative images of mucosal and bone tissue in the negative control and composite groups at ×40 magnification. **(C,D)** Representative images of bone tissue in the negative control and composite groups at ×100 magnification. **(E)** Quantitative analysis of empty osteocyte lacunae percentage in bone tissue. Data for the model control and Bio-Oss granules groups were previously reported (Shi et al., 2025) and are included here for comparison. ***, *P* < 0.001 vs. negative control group; ◆◆◆, *P* < 0.001 among the model control, Bio-Oss granules, and composite groups (one-way ANOVA with Bonferroni *post hoc* test).

Histological analysis revealed that the mucosa in the negative control group was intact (Figure 9A). In contrast, both the model control and Bio-Oss granules groups exhibited incomplete mucosa with collapse of the epithelial and connective tissues, as previously reported (Shi et al., 2025). The composite group showed continuous and well-healed mucosal coverage (Figure 9B). Quantitative analysis of empty osteocyte lacunae demonstrated that the negative control group had the lowest percentage (6.00% ± 1.90%), which was significantly lower than those of the other three groups (*P* < 0.001). The composite group (20.67% ± 2.16%) exhibited a significantly lower percentage of empty osteocyte lacunae than both the model control group (80.67% ± 3.14%) and the Bio-Oss granules group (80.33% ± 1.63%, with *P* < 0.001), while no significant difference was observed between the latter two groups (*P* > 0.05, Figure 9E).

## 4 Discussion

The present study evaluated the preventive efficacy of the hucMSCs/Bio-Oss granules composite against MRONJ. The composite group showed significantly improved healing compared with both the model control and Bio-Oss granules groups, based on macroscopic, radiological, and histological assessments. These findings support our hypothesis that the hucMSC/Bio-Oss granules composite enhances tissue regeneration under BP-treated conditions. However, healing in the composite group remained inferior to that in the negative control group, suggesting a substantial improvement toward normal levels, although complete restoration was not achieved. In addition, no significant difference was observed between the model control and Bio-Oss granules groups.

When comparing the BP-treated groups with the negative control group, distinct MRONJ-like lesions were observed following BP exposure, characterized by mucosal discontinuity, bone exposure, and pathological changes consistent with impaired healing. In contrast, the negative control group, which was not exposed to BP, showed normal mucosal and bone healing, serving as a physiological control. Among the BP-treated rats, particularly those in the model control group and the Bio-Oss granules group, mucosal breakdown and bone exposure were frequently observed, accompanied by marked bone resorption and a significantly higher proportion of empty osteocyte lacunae. These pathological, radiological, and histological findings are consistent with the typical features of MRONJ reported in previous animal studies (Yan et al., 2022), suggesting that in the present study, BP administration combined with tooth extraction successfully reproduced an MRONJ-like phenotype in rats. It is noteworthy that, although the composite group achieved a certain degree of preventive efficacy, the healing outcomes did not reach those observed in the negative control group, and significant differences remained across multiple parameters. The remaining differences may reflect the persistent effects of BP exposure on bone turnover and angiogenesis, along with the limited capacity of cell-based interventions to fully reverse the compromised microenvironment due to factors such as the survival and functional activity of transplanted hucMSCs *in vivo*. Additionally, BP-induced alterations in the local immune microenvironment may further disrupt osteoclast-related signaling and bone remodeling processes (Jiang et al., 2023), thereby impairing tissue repair and contributing to incomplete healing compared with physiological conditions.

DAPI staining and nucleic acid quantification in this study revealed low but detectable levels of DNA and RNA in Bio-Oss granules, verifying the effectiveness of the deproteinization process used in the manufacturing of this material (Schwartz et al., 2000) and ensuring the reliability of subsequent gene expression analysis. These low levels likely represent trace fragmented residues or assay background signals rather than substantial nucleic acid content intrinsic to the material. In addition, the present study demonstrated enhanced osteogenic differentiation of hucMSCs cultured with Bio-Oss granules, as evidenced by increased expression of ALP, OPN, and RUNX2, which is consistent with previous studies (Yu et al., 2014; Bae et al., 2019). Bio-Oss is generally recognized as an osteoconductive rather than an osteoinductive material (Jensen et al., 2006; Traini et al., 2007). However, Schwartz et al. (2000) have proposed that trace amounts of osteogenic growth factors, such as bone morphogenetic protein-2 and transforming growth factor-β, may remain bound to the hydroxyapatite crystals even after deproteinization, potentially endowing the material with weak osteoinductive properties. Although this hypothesis remains controversial, it may partially account for the enhanced osteogenic gene expression observed in our co-culture experiments, suggesting that the physicochemical structure of Bio-Oss and its possible residual signaling molecules can synergistically modulate stem cell behavior and contribute to the osteogenic potential observed when combined with hucMSCs.

In the present study, Bio-Oss granules alone showed no preventive effect against MRONJ, with healing outcomes that were not significantly different from those of the model control group. This finding is consistent with the study by Zang et al. (2019), which used coral-derived HA in an MRONJ-like rabbit model. Both Bio-Oss granules and coral-derived HA share similar physicochemical characteristics, as they are calcium phosphate-based, biocompatible and osteoconductive materials (Jensen et al., 2006; Miron et al., 2024). Under BP-compromised conditions, where osteoclastic and osteoblastic activities are suppressed and angiogenesis is impaired (Allegra et al., 2010; Abe et al., 2012; Patntirapong et al., 2012), either material alone is insufficient to achieve effective bone regeneration. In addition, MSCs have also been reported to regulate osteoclastogenesis and bone remodeling, both of which are critically impaired in MRONJ (He et al., 2017). Furthermore, Bio-Oss granules are slowly resorbable materials that tend to remain within the defect sites for prolonged periods (Miron et al., 2024). In the necrotic and low-turnover environment of MRONJ, such persistence may further impede bone remodeling and soft-tissue integration, thereby contributing to delayed or incomplete healing (Hibi, 2020).

Notably, rats in the composite group exhibited substantial improvement, with no bone exposure and a higher rate of complete mucosal coverage, as well as significantly enhanced bone regeneration compared with the other BP-treated groups, indicating that the addition of hucMSCs effectively mitigated the deleterious effects of BP exposure by promoting both mucosal healing and bone formation. The qRT-PCR results further supported these findings, showing that co-culture with Bio-Oss granules significantly upregulated the expression of osteogenic genes (RUNX2, ALP, OPN) in hucMSCs, while maintaining high expression of the stemness-related gene OCT4. These results indicate that Bio-Oss granules could provide a supportive microenvironment that promotes hucMSCs proliferation and differentiation and may help maintain their paracrine potential, thereby enabling them to exert paracrine effects (Dong et al., 2021; Zheng et al., 2024). Such preservation of hucMSCs viability and multipotency is essential for the sustained release of paracrine factors that regulate epithelial cell migration and accelerate mucosal closure, ultimately contributing to MRONJ prevention (Zang et al., 2019). In this context, the low degradation rate of Bio-Oss may help maintain structural stability, which in turn facilitates hucMSCs adhesion, proliferation, and migration for new bone deposition. Therefore, the synergistic interaction between hucMSCs and the Bio-Oss scaffold—combining cellular bioactivity, paracrine regulation, and structural support—accounts for the superior preventive efficacy observed in the composite group, underscoring the therapeutic potential of MSC-based biomaterial composites for MRONJ prevention.

From a translational perspective, these findings suggest potential clinical implications for MRONJ prevention. Bio-Oss is already widely used in oral and maxillofacial surgery as a bone substitute, and its combination with hucMSCs may represent a feasible strategy to enhance bone regeneration and mucosal healing in patients at risk of MRONJ following tooth extraction. These results provide preliminary experimental evidence supporting the potential application of MSC-based biomaterial composites for MRONJ prevention. Importantly, integrating a clinically applicable scaffold with a readily accessible MSC source may offer a more feasible pathway toward clinical translation.

Although this study provides valuable insights into the preventive potential of the hucMSCs/Bio-Oss granules composite, the experiments were conducted in a rat model with a relatively short observation period, which may not fully represent the long-term and multifactorial nature of human MRONJ. Moreover, although *in vitro* results demonstrated the osteogenic differentiation potential of hucMSCs, no *in vivo* cell tracking was performed, and thus the survival and differentiation of transplanted cells could not be determined. In addition, while our findings suggest possible mechanisms involving osteogenic potential and paracrine modulation, angiogenic and inflammatory markers were not evaluated, and the specific molecular pathways were not directly examined. These aspects warrant further investigation in future studies with extended follow-up, cell tracking approaches, and more detailed mechanistic analyses.

## 5 Conclusion

Bio-Oss granules alone showed limited efficacy in preventing MRONJ in BP-treated rats. In contrast, the hucMSCs/Bio-Oss granules composite effectively prevented MRONJ development while promoting mucosal healing and new bone formation. These findings support the potential of the hucMSCs/Bio-Oss granules composite as a preventive strategy for MRONJ.

## Data Availability

The original contributions presented in the study are included in the article/supplementary material, further inquiries can be directed to the corresponding author.
